# Exercise-induced change in FGF21 and adiponectin and their association with metabolic syndrome in older women: a randomized controlled trial

**DOI:** 10.1016/j.jnha.2026.100923

**Published:** 2026-07-04

**Authors:** Liangliang Li, Deokhwa Jeong, Hyeongmo Jeong, Jiye Choi, Sunghwun Kang

**Affiliations:** a College of Physical Education, Sichuan Agricultural University, Ya’an, China; b Department of Smart Health Science and Technology, Kangwon National University, Gangwon-do, Republic of Korea; c Department of Sport Science, Kangwon National University, Gangwon-do, Republic of Korea

**Keywords:** Fibroblast growth factor 21–adiponectin axis, Metabolic health, Aging, Endocrine regulation, Physical activity

## Abstract

**Background:**

Metabolic syndrome is common in older adults and increases cardiometabolic risk. The fibroblast growth factor 21 (FGF21)–adiponectin axis regulates metabolic homeostasis, but its exercise responsiveness may differ by metabolic status. This study investigated the effects of exercise on circulating FGF21, adiponectin, and the FGF21/adiponectin ratio in older women with and without metabolic syndrome.

**Methods:**

Sixty older women (75.2 ± 4.3 years) were classified by metabolic syndrome status and randomly assigned to control or exercise groups. Exercise groups completed a 10-week supervised combined exercise program consisting of body-weight resistance and cross-linked elastic resistance band training (3 sessions/week, 60 min/session), while controls maintained their usual lifestyle. Blood biomarkers, body composition, physical fitness, and metabolic parameters were assessed before and after the intervention and analyzed using two-way repeated-measures ANOVA.

**Results:**

The FGF21–adiponectin axis was modulated by the intervention. Adiponectin increased in both exercise groups (NMSE: mean change, 1.23; 95% CI, 0.98–1.49; d = 1.04; MSE: mean change, 0.86; 95% CI, 0.31–1.40; d = 0.71). FGF21 increased in the NMSE group (mean change, 16.75; 95% CI, 5.46–28.05; d = 0.40) but decreased in the MSE group (mean change, −15.91; 95% CI, −28.35 to −3.48; d = −0.33). The FGF21/adiponectin ratio decreased in both exercise groups, with a greater reduction in MSE (NMSE: mean change, −2.18; 95% CI, −4.18 to −0.18; d = −0.29; MSE: mean change, −6.48; 95% CI, −9.82 to −3.15; d = −0.86). Secondary outcomes, including body composition, physical fitness, lipid profiles, insulin resistance markers, and inflammatory markers, also improved with small-to-large effect sizes.

**Conclusion:**

Combined exercise improved metabolic health and modulated the FGF21–adiponectin axis in older women, with differential FGF21 responses by metabolic status. The reduced FGF21/adiponectin ratio may indicate exercise-induced metabolic adaptation.

**Trial registration:**

KCT 0011558.

## 1 Introduction

Metabolic syndrome is a complex metabolic disorder characterized by insulin resistance, chronic low-grade inflammation, and dysregulated lipid metabolism, and it substantially increases the risk of obesity, type 2 diabetes, and cardiovascular disease [1]. Its prevalence is particularly high among older adults because of aging-related physiological changes and reduced physical activity, making it a major public health concern [2,3]. Notably, Asian populations, including Koreans, exhibit a relatively high prevalence of metabolic syndrome despite lower body mass index compared with Western populations, which has been attributed to greater visceral adiposity and metabolic susceptibility [4]. Therefore, identifying mechanisms and effective strategies to improve metabolic health in older adults is critical for chronic disease prevention.

The fibroblast growth factor 21 (FGF21)–adiponectin axis has emerged as an important pathway regulating metabolic homeostasis [5]. FGF21, primarily secreted by the liver and skeletal muscle, plays a key role in glucose and lipid metabolism and insulin sensitivity, whereas adiponectin exerts anti-inflammatory and insulin-sensitizing effects across multiple organs [6]. FGF21 stimulates adiponectin secretion, and adiponectin mediates downstream metabolic effects [7,8]. However, under metabolic dysfunction such as metabolic syndrome, impaired FGF21 signaling leads to elevated circulating FGF21 and reduced adiponectin levels [[9], [10], [11]]. Accordingly, the FGF21/adiponectin ratio has been proposed as a potential biomarker of metabolic dysfunction [12,13].

Exercise is an effective non-pharmacological strategy for improving metabolic health and may modulate the FGF21–adiponectin axis [[14], [15], [16]]. However, exercise-induced responses appear to differ according to metabolic status, with contrasting patterns reported between individuals with and without metabolic disorders [17]. Despite this, evidence remains limited, particularly in older women. Therefore, this study aimed to examine the effects of a 10-week supervised combined exercise program consisting of body-weight resistance and cross-linked elastic resistance band training (CLX training) on circulating FGF21, adiponectin, and the FGF21/adiponectin ratio in older women according to metabolic syndrome status. We hypothesized that this combined exercise program would increase circulating adiponectin levels and reduce the FGF21/adiponectin ratio in older women, with differential FGF21 responses according to metabolic syndrome status.

## 2 Methods

### 2.1 Study design & participation

This study was conducted in accordance with the Declaration of Helsinki and approved by the Institutional Review Board (IRB) of Kangwon National University (KWNUIRB-2022-08-003). The study was retrospectively registered in the Korea Clinical Trials Registry (KCT0011558; January 31, 2026), and written informed consent was obtained from all participants. Sample size was calculated using G*Power software (version 3.1.9.7; Heinrich Heine University, Düsseldorf, Germany) with an assumed medium effect size (f = 0.25), α = 0.05, and statistical power of 0.80. The minimum required sample size was 48 participants, and more than 60 participants were initially recruited considering an approximate 20% dropout rate.

Participants were screened according to predefined eligibility criteria. Inclusion criteria were as follows: older women who met the classification criteria for metabolic syndrome status, were physically able to complete the 10-week exercise program, had not participated in regular structured exercise within the previous 3 months, and voluntarily agreed to participate. Exclusion criteria included musculoskeletal disorders or medical conditions contraindicating exercise participation, participation in another clinical trial within the previous 4 weeks, use of medications affecting metabolic function or body weight regulation, severe or unstable diseases, or any condition limiting regular exercise participation.

Prior to baseline assessment, waist circumference, triglycerides (TG), blood pressure, and fasting blood glucose were measured. In this study, metabolic syndrome was diagnosed according to the criteria established by the Korean Society of Cardiovascular and Metabolic Syndrome (KSCMS) [18]. Specifically, metabolic syndrome was defined as the presence of three or more of the following five components: abdominal obesity (waist circumference ≥90 cm for men and ≥85 cm for women), elevated triglycerides (TG ≥ 150 mg/dL), reduced high-density lipoprotein cholesterol (HDL-C <40 mg/dL for men and <50 mg/dL for women), elevated blood pressure (systolic blood pressure ≥ 130 mmHg or diastolic blood pressure ≥85 mmHg, or current use of antihypertensive medication), and elevated fasting glucose (fasting glucose ≥100 mg/dL or current use of glucose-lowering medication). Based on these criteria, a total of 60 older women voluntarily participated in the study and were classified into two groups: a metabolic syndrome group (n = 30) and a non-metabolic syndrome group (n = 30).

The study design and baseline participant characteristics are presented in Supplementary Fig. S1 and Table S1.

All participants completed baseline (Pre) and post-intervention (Post, 10 weeks) assessments at the Exercise Physiology Laboratory, Department of Sports Science, Kangwon National University, following an overnight fast. Participants in both the metabolic syndrome and non-metabolic syndrome groups were randomly assigned by drawing a paper labeled “A” or “B” from an opaque box. Participants who drew “A” were allocated to the control group, whereas those who drew “B” were assigned to the exercise group. Consequently, participants were allocated to the metabolic syndrome control group (MSC, n = 15), metabolic syndrome exercise group (MSE, n = 15), non-metabolic syndrome control group (NMSC, n = 15), or non-metabolic syndrome exercise group (NMSE, n = 15). During the study period, 12 participants withdrew from the study, including three from MSC, two from MSE, four from NMSC, and three from NMSE.

**Fig. 1:**
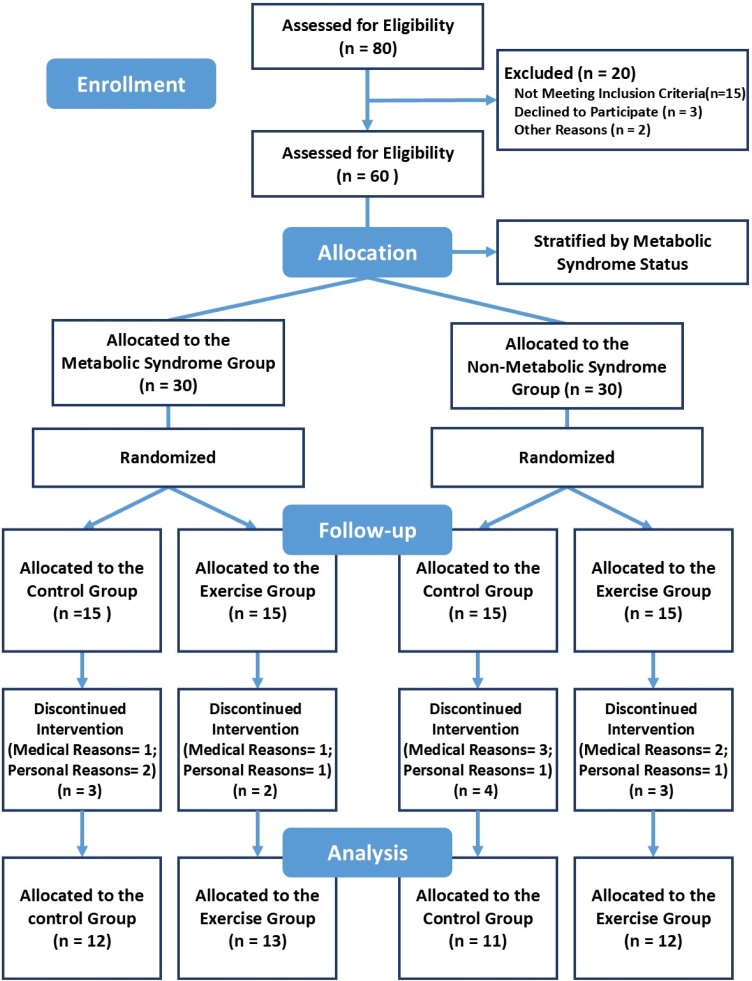
CONSORT participant flow diagram.

The CONSORT flow diagram illustrating participant progression throughout the study is presented in Fig. 1.

### 2.2 Outcome measures

The primary outcomes of this study were circulating adiponectin, FGF21, and the FGF21/adiponectin ratio. The FGF21/adiponectin ratio was calculated individually for each participant using the measured serum concentrations and then averaged at the group level. Secondary outcomes included inflammatory cytokines, lipid profiles, insulin resistance markers, body composition, blood pressure, and physical fitness.

### 2.3 Hematological analysis

Venous blood samples were collected from all participants at baseline and after the 10-week intervention period. Blood samples were obtained from the antecubital vein at 08:00 following a 12-h overnight fast and adequate rest, while minimizing physical activity. To minimize acute exercise effects, post-intervention blood samples were collected at least 48 h after the last exercise session. All venipuncture procedures were performed by a licensed nurse.

Whole blood samples were collected in serum separator tubes, allowed to clot at room temperature for 30 min, and centrifuged at 3,500 g for 10 min. The separated serum samples were stored at −80 °C until further analysis. Fasting glucose was measured using the OneTouch Ultra system (LifeScan, Milpitas, CA, USA), and serum fasting insulin was measured using a commercially available assay kit (440132, Beckman Coulter, Brea, CA, USA). Insulin resistance was assessed using the homeostasis model assessment of insulin resistance (HOMA-IR): fasting insulin [μIU/mL] × fasting glucose [mmol/L] / 22.5 [19].

Serum concentrations of FGF21 and adiponectin (DY2537 and DY1065; R&D Systems, Minneapolis, MN, USA), as well as inflammatory cytokines including interleukin-1β (IL-1β), interleukin-6 (IL-6), and tumor necrosis factor-α (TNF-α) (DY201, DY206, and DY210; R&D Systems, Minneapolis, MN, USA), were quantified using enzyme-linked immunosorbent assay (ELISA) kits according to the manufacturer’s instructions. Serum lipid profiles, including high-density lipoprotein cholesterol (HDL-C), total cholesterol (TC), and triglycerides (TG), were measured using commercially available assay kits (ab65390, ab65359, and ab65336; Abcam Plc, Cambridge, UK). Low-density lipoprotein cholesterol (LDL-C) was calculated using the Friedewald equation: LDL-C = TC − HDL-C − (TG/5).

### 2.4 Assessment of body composition

Body composition variables were measured using a bioelectrical impedance analyzer (BIA) (Inbody 720 Body Composition Analyzer, Biospace, Seoul, Republic of Korea). Participants were assessed barefoot and in light clothing, after removing shoes, socks, and heavy accessories. Weight (kg), fat mass (kg), and muscle mass (kg) were recorded to the nearest 0.1 kg. Percent body fat was calculated based on impedance values obtained through multiple frequencies. Body mass index (BMI) was determined as weight in kilograms divided by height in meters squared (kg/m^2^).

### 2.5 Blood pressure

Blood pressure was measured after participants had rested in a seated position for at least 5 min using an automated oscillometric blood pressure monitor (BPBIO330, Biospace, Seoul, Korea). Measurements were obtained from the right upper arm supported at heart level under identical conditions at baseline and after the intervention period. Systolic blood pressure (SBP) and diastolic blood pressure (DBP) were measured twice at each time point by a licensed nurse, and the mean value was used for analysis.

### 2.6 Assessment of the senior fitness test

Physical fitness was assessed using the Senior Fitness Test (SFT), which has been previously developed and validated [20]. All participants completed the SFT at baseline and after week 10. The SFT evaluated muscle strength, muscular endurance, flexibility, balance, cardiorespiratory endurance, and coordination. Muscle strength was measured using a digital hand grip dynamometer (BS-HG, Biospace, Korea), with grip strength tested twice per hand and the best value from each hand averaged. Muscular endurance was assessed using the 30-second chair stand test, in which participants repeatedly stood up and sat down from a 40 cm chair with their arms crossed over the chest. Flexibility was assessed using the sit-and-reach test with a digital forward flexion meter (BS-FF, Biospace, Korea). Balance was assessed using the Timed Up and Go (TUG) test, and coordination was assessed using the Figure-of-8 Walk Test (F8WT). Cardiorespiratory endurance was assessed using the 2-minute step test, in which only steps reaching the required knee height were counted. Strength and flexibility tests were performed twice, and the higher value was recorded.

All SFT evaluations were administered by a certified health and exercise specialist.

### 2.7 Exercise intervention

The exercise program consisted of a 10-week progressive combined exercise intervention based on body-weight training and cross-linked elastic resistance band training (CLX training). The program was designed in accordance with exercise prescription guidelines for older adults established by the American College of Sports Medicine (ACSM) and the National Strength and Conditioning Association (NSCA) [21]. The intervention was performed three times per week for 60 min per session and consisted of three phases: warm-up, main exercise, and cool-down.

The warm-up phase lasted approximately 10 min and included light walking and dynamic stretching. The main exercise phase lasted approximately 40 min and consisted of resistance exercises using body-weight training and CLX training. The cool-down phase lasted approximately 10 min and included light walking and static stretching. The primary method for controlling exercise intensity during the resistance-based components was the rating of perceived exertion (RPE), whereas heart rate was used as a supplementary monitoring variable to assess overall physiological response, energy expenditure, and exercise safety.

All participants wore a wireless heart rate monitor (Polar M400, Polar Electro Oy, Finland) during each session. Exercise intensity was progressively controlled using RPE, with target ranges of 11–12 during weeks 1–3, 13–14 during weeks 4–6, and 14–15 during weeks 7–10. The target energy expenditure for each session was approximately 300–350 kcal, and heart rate was monitored for safety and overall physiological monitoring rather than as the primary index for resistance exercise intensity prescription.

Detailed information regarding the exercise intervention can be found in Table 1.

**Table 1:** Exercise intervention protocol.

Variable parameter	Training protocol	Duration (min)	Volume	Intensity	Frequency
Warm-up	Dynamic Stretching	10	–	1 ∼ 3 weeks 11 ∼ 12 RPE 4 ∼ 6 weeks 13 ∼ 14 RPE 7 ∼ 10 weeks 14 ∼ 15 RPE 300 ∼ 350 kcal HRmax 60 ∼ 80 %	10 weeks (3 times per week) 30 sessions
Body weight	Wall Push-up, Wall Angels, Lunges, Squats, Marching in Place, Single-Leg Stance, and Side Stepping	20	Each drill: 3 set * 40 seconds		
CLX training	Band Pull Apart, Front Raise, Lateral Raise, Shoulder Press, Biceps Curl, Triceps Extension, Squat and Bent-over Row	20	Each drill: 3 set * 10 reps		
Cool down	Static Stretching	10	–		

2.8. Lifestyle advice

All participants were instructed to maintain their habitual daily routines and to avoid initiating any new structured exercise that could potentially impact the study outcomes. Participants were also requested not to implement major dietary modifications that might influence body composition. Although no specific software was used to record dietary intake or daily physical activity, compliance was monitored through weekly self-reports and regular group text message reminders throughout the intervention period to minimize potential confounding effects. Additionally, all participants signed a written agreement confirming that they would not participate in any other exercise, weight loss, or health-related programs that could influence the study outcomes, and that they would adhere to the investigators’ instructions throughout the trial.

### 2.8 Statistical analysis

All results are presented as mean ± standard deviation. Statistical analyses were performed with SPSS version 29.0 (SPSS Inc., Chicago, IL, USA). An initial two-way repeated measures analysis of variance (ANOVA) was conducted to assess the main effects of group, time, and their interaction. If significant interaction effects were found, post-hoc analyses involved Bonferroni-adjusted pairwise comparisons for between-group differences and paired-sample t-tests to compare within-group differences over time. All statistical tests were two-tailed. Statistical significance was defined as α = 0.05. Non-significant trends are described up to p = 0.1 in the text.

Effect sizes were calculated using Cohen’s d to quantify the magnitude of intervention effects. First, the mean difference between post- and pre-intervention values was calculated by subtracting the pre-intervention mean from the post-intervention mean. The pooled standard deviation was then computed as the square root of the average of the squared pre- and post-intervention standard deviations. Finally, Cohen’s d was obtained by dividing the mean difference by the pooled standard deviation. Effect sizes were interpreted as small (d < 0.2), medium (d ≥ 0.5), and large (d ≥ 0.8) according to conventional criteria [22].

## 3 Results

### 3.1 Changes in adiponectin, FGF21, the FGF21/adiponectin ratio and inflammatory markers

**Fig. 2:**
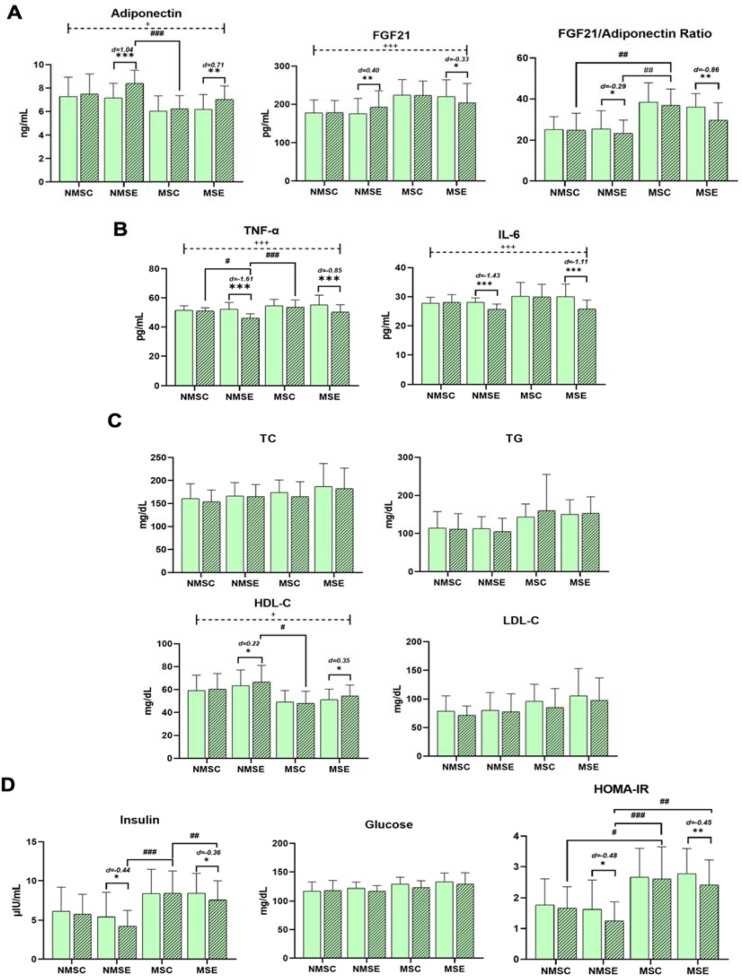
(A) Changes in Adiponectin, FGF21, and FGF21/Adiponectin Ratio. (B) Changes in Inflammatory Markers. (C) Changes in blood lipid profiles. (D) Changes in insulin resistance markers. Values are expressed as mean ± SD. NMSC, non-metabolic syndrome control group; NMSE, non-metabolic syndrome exercise group; MSC, metabolic syndrome control group; MSE, metabolic syndrome exercise group; FGF21, fibroblast growth factor 21; TNF-α, tumor necrosis factor-alpha; IL-6, interleukin-6; HOMA-IR, homeostasis model assessment of insulin resistance; TC, total cholesterol; TG, triglycerides; HDL-C, high-density lipoprotein cholesterol; LDL-C, low-density lipoprotein cholesterol. Data were analyzed by two-way repeated-measures ANOVA. ^+^p < 0.05, ^++^p < 0.01, and ^+++^p < 0.001 indicate a significant group × time interaction; ^#^p < 0.05, ^##^p < 0.01, and ^###^p < 0.001 indicate a significant difference between groups; *p < 0.05, **p < 0.01, and ***p < 0.001 indicate a significant within-group change over time. Effect sizes were calculated using Cohen’s d and interpreted as small (d < 0.2 and < 0.5), medium (d ≥ 0.5 and < 0.8), and large (d ≥ 0.8) to evaluate practical significance.

Results are presented in Fig. 2A–B. Two-way repeated-measures ANOVA showed significant group × time interactions for adiponectin (F = 3.768, p = 0.017), FGF21 (F = 6.551, p < 0.001), TNF-α (F = 10.183, p < 0.001), and IL-6 (F = 15.656, p < 0.001). Significant main effects of group and time were observed for adiponectin and TNF-α (all p < 0.05), whereas only a main effect of time was observed for IL-6 (p < 0.001). For FGF21, a significant main effect of group was observed (F = 3.543, p = 0.022), with no significant time effect.

Post hoc analyses showed significant changes, with Cohen’s d effect sizes indicating the magnitude of these changes. Adiponectin significantly increased in both exercise groups (NMSE: p < 0.001, d = 1.04; MSE: p < 0.01, d = 0.71), whereas FGF21 responses differed by metabolic status, increasing in NMSE (p < 0.01, d = 0.40) but decreasing in MSE (p < 0.05, d = −0.33). Inflammatory markers, including TNF-α and IL-6, significantly decreased in both exercise groups (NMSE and MSE) (all p < 0.001, d = −1.61 to −0.85).

For the FGF21/adiponectin ratio, significant main effects of group (F = 9.114, p < 0.001) and time (F = 9.996, p = 0.003) were observed, with no significant interaction. The FGF21/adiponectin ratio was calculated individually for each participant and then averaged at the group level. The ratio was lower in the NMSC and NMSE groups than in the MSC group and significantly decreased in both exercise groups (NMSE: p < 0.05, d = −0.29; MSE: p < 0.01, d = −0.86). To clarify the magnitude of change, percentage changes were descriptively examined. In the NMSE group, adiponectin increased by 17.2%, whereas FGF21 increased by 9.5%, resulting in a 13.9% reduction in the FGF21/adiponectin ratio. In the MSE group, adiponectin increased by 14.0%, whereas FGF21 decreased by 7.2%, producing a descriptively greater reduction in the FGF21/adiponectin ratio (−26.5%).

### 3.2 Changes in blood lipid profiles and insulin resistance markers

Results for blood lipid profiles and insulin resistance markers are presented in Fig. 2C-D. Two-way repeated-measures ANOVA showed a significant group × time interaction for HDL-C (F = 2.857, p = 0.048). Significant main effects of group were observed for triglycerides (TG; F = 3.810, p = 0.016), HDL-C (F = 4.643, p = 0.007), insulin (F = 5.194, p = 0.004), and HOMA-IR (F = 6.885, p < 0.001). Significant main effects of time were found for HDL-C (F = 6.595, p = 0.014), LDL-C (F = 6.811, p = 0.012), insulin (F = 7.871, p = 0.007), glucose (F = 4.498, p = 0.040), and HOMA-IR (F = 8.630, p = 0.005).

Post hoc analyses with Cohen’s d effect sizes showed that HDL-C increased significantly in the NMSE (p < 0.05, d = 0.22) and MSE (p < 0.05, d = 0.35) groups. Insulin and HOMA-IR decreased significantly in both exercise groups (NMSE: p < 0.05, d = −0.44 and −0.48; MSE: p < 0.05 to 0.01, d = −0.36 and −0.45, respectively). Additionally, insulin levels were lower in the NMSE group than in the MSC and MSE groups, and HOMA-IR values were lower in the NMSC and NMSE groups than in the MSC group, with NMSE showing lower values than MSE.

No significant effects were observed for total cholesterol (TC), triglycerides (time or interaction), LDL-C (group or interaction), or glucose (group or interaction) (all p > 0.05).

### 3.3 Changes in body composition and blood pressure

Results for body composition and blood pressure are presented in Table 2. Two-way repeated-measures ANOVA showed significant group × time interactions for body fat mass (F = 2.943, p = 0.043) and percent body fat (F = 2.986, p = 0.041). Significant main effects of group were observed for body fat mass (F = 7.950, p < 0.001), percent body fat (F = 8.919, p < 0.001), and waist circumference (F = 4.936, p = 0.005), whereas significant main effects of time were found for percent body fat (F = 4.394, p = 0.042), muscle mass (F = 6.007, p = 0.018), and waist circumference (F = 4.874, p = 0.033).

**Table 2:** Change in body composition and blood pressure.

Variable	Group	Baseline (0 week)	Post (10 weeks)	Cohen’d	F-Value (*p*-Value)	Post-Hoc
Weight (kg)	NMSC(n = 11) ^a^	56.87 ± 3.77	57.16 ± 4.07		G:2.54 (0.069)	
	NMSE(n = 12) ^b^	56.80 ± 7.43	57.03 ± 6.84		T:3.547 (0.066)	
	MSC(n = 12) ^c^	61.21 ± 6.21	61.57 ± 6.07		G × T: 0.040 (0.989)	
	MSE(n = 13) ^d^	61.67 ± 5.36	61.92 ± 5.81			
Muscle mass (kg)	NMSC(n = 11) ^a^	19.34 ± 2.65	19.43 ± 1.97		G: 1.716 (0.178)	
	NMSE(n = 12) ^b^	**19.59 ± 2.19**	20.27 ± 2.73*	**0.27**	**T: 6.007 (0.018)**	
	MSC(n = 12) ^c^	17.91 ± 2.64	17.98 ± 2.43		G × T: 1.234 (0.309)	
	MSE(n = 13) ^d^	**18.38 ± 1.97**	18.86 ± 2.07*	**0.24**		
Body fat mass (kg)	NMSC(n = 11) ^a^	17.03 ± 4.50	16.65 ± 4.51		**G: 7.950(<0.001)**	a,b < c,d
	NMSE(n = 12) ^b^	**16.70 ± 4.06**	15.88 ± 3.82*	**−0.21**	T: 1.985(0.166)	
	MSC(n = 12) ^c^	22.04 ± 3.71	22.73 ± 3.67		**G × T: 2.943(0.043)**	
	MSE(n = 13) ^d^	**22.75 ± 4.64**	22.16 ± 4.67*	**−0.13**		
Precent body fat (%)	NMSC(n = 11) ^a^	29.66 ± 6.50	28.85 ± 6.31		**G: 8.919(<0.001)**	a,b < c,d
	NMSE(n = 12) ^b^	**29.10 ± 4.77**	27.58 ± 4.80*	**−0.32**	**T: 4.394(0.042)**	
	MSC(n = 12) ^c^	35.88 ± 3.75	36.77 ± 3.48		**G × T: 2.986(0.041)**	
	MSE(n = 13) ^d^	**36.66 ± 5.38**	35.52 ± 5.25*	**−0.21**		
BMI (kg/m^2^)	NMSC(n = 11) ^a^	24.88 ± 1.97	24.81 ± 2.23		G: 2.001(0.128)	
	NMSE(n = 12) ^b^	24.61 ± 2.46	24.68 ± 2.25		T: 1.559(0.218)	
	MSC(n = 12) ^c^	26.27 ± 2.48	26.73 ± 2.85		G × T: 1.338(0.274)	
	MSE(n = 13) ^d^	26.54 ± 2.88	26.57 ± 3.00			
WC (cm)	NMSC(n = 11) ^a^	80.41 ± 7.77	80.60 ± 7.74		**G: 4.936(0.005)**	b < c
	NMSE(n = 12) ^b^	**80.08 ± 6.17**	79.16 ± 6.52*	**−0.14**	**T: 4.874(0.033)**	
	MSC(n = 12) ^c^	88.11 ± 6.91	87.78 ± 8.08		G × T: 1.419(0.250	
	MSE(n = 13) ^d^	**88.05 ± 6.33**	86.94 ± 6.26*	**−0.18**		
SBP (mmHg)	NMSC(n = 11) ^a^	131.55 ± 14.84	132.82 ± 19.13		G: 2.399(0.081)	
	NMSE(n = 12) ^b^	136.67 ± 12.21	139.58 ± 16.09		T: 0.135(0.715)	
	MSC(n = 12) ^c^	145.50 ± 9.60	141.92 ± 16.71		G × T: 0.462(0.710)	
	MSE(n = 13) ^d^	142.00 ± 12.54	144.69 ± 10.80			
DBP (mmHg)	NMSC(n = 11) ^a^	65.64 ± 8.76	70.09 ± 8.65		G: 1.121(0.351)	
	NMSE(n = 12) ^b^	70.67 ± 10.14	76.67 ± 9.03		**T: 9.204(0.004)**	
	MSC(n = 12) ^c^	69.08 ± 8.39	73.08 ± 6.97		G × T: 0.206(0.892)	
	MSE(n = 13) ^d^	71.92 ± 14.65	74.85 ± 10.24			

Values are presented as Mean ± SD. a = NMSC non-metabolic syndrome control group; b = NMSE, non-metabolic syndrome exercise group; c = MSC, metabolic syndrome control group; d = MSE, metabolic syndrome exercise group; WC, waist circumference; BMI, body mass index; SBP, systolic blood pressure; DBP, diastolic blood pressure. Statistical analysis was performed using the paired t-test: *p < .05; ***p < .001. Effect sizes cohen’d were interpreted small (d < 0.2), medium (d ≥ 0.5), and large (d ≥ 0.8).

Post hoc analyses indicated that body fat mass and percent body fat were lower in the NMSC and NMSE groups than in the MSC and MSE groups. Paired t-test analyses with Cohen’s d effect sizes showed that body fat mass and percent body fat significantly decreased in the NMSE (p < 0.05, d = −0.21 and −0.32, respectively) and MSE (p < 0.05, d = −0.13 and −0.21, respectively) groups. Muscle mass significantly increased in the NMSE (p < 0.05, d = 0.27) and MSE (p < 0.05, d = 0.24) groups, whereas waist circumference significantly decreased in the NMSE (p < 0.05, d = −0.14) and MSE (p < 0.05, d = −0.18) groups. Additionally, waist circumference was significantly lower in the NMSE group than in the MSC group. No significant changes were observed for weight or BMI (all p > 0.05).

For blood pressure, no significant group × time interactions were observed for systolic or diastolic blood pressure (all p > 0.05). A significant main effect of time was found for diastolic blood pressure (F = 9.204, p = 0.004), with no significant group effect (p = 0.351). No significant effects were observed for systolic blood pressure.

### 3.4 Changes in senior fitness test

Results for the senior fitness test are presented in Table 3. Two-way repeated-measures ANOVA showed a significant group × time interaction for the 30-second chair stand (F = 3.594, p = 0.021). Significant main effects of time were observed for grip strength (left: F = 27.255, p < 0.001; right: F = 12.858, p < 0.001), 30-second chair stand (F = 7.831, p = 0.008), 2-minute step (F = 20.088, p < 0.001), Timed Up and Go (TUG; F = 28.220, p < 0.001), and Figure-of-8 Walk Test (F8WT; F = 19.723, p < 0.001). No significant main effects of group were observed (all p > 0.05).

**Table 3:** Change in senior fitness test.

Variable	Group	Baseline (0 week)	Post (10 weeks)	Cohen’d	F-Value (*p*-Value)	Post-Hoc
Grip strength (Rt) (kg)	NMSC(n = 11) ^a^	23.72 ± 4.03	24.28 ± 3.52		G: 0.116 (0.950)	
	NMSE(n = 12) ^b^	**23.03 ± 4.00**	24.84 ± 3.82*	**0.46**	**T: 12.858 (<0.001)**	
	MSC(n = 12) ^c^	22.68 ± 4.57	23.56 ± 4.90		G × T: 0.715 (0.548)	
	MSE(n = 13) ^d^	**22.93 ± 4.34**	24.15 ± 4.49*	0.28		
Grip strength (Lt) (kg)	NMSC(n = 11) ^a^	21.31 ± 3.21	22.66 ± 3.88		G: 0.783 (0.510)	
	NMSE(n = 12) ^b^	21.28 ± 3.77	23.98 ± 3.71**	0.72	**T: 27.255 (<0.001)**	
	MSC(n = 12) ^c^	19.57 ± 4.22	20.73 ± 3.60		G × T: 1.025 (0.391)	
	MSE(n = 13) ^d^	20.39 ± 5.57	22.48 ± 5.40***	0.38		
30 sec chair stand (rep)	NMSC(n = 11) ^a^	20.18 ± 4.88	19.18 ± 4.31		G: 2.309 (0.089)	
	NMSE(n = 12) ^b^	**21.67 ± 4.12**	26.08 ± 7.04*	**0.76**	**T: 7.831 (0.008)**	
	MSC(n = 12) ^c^	18.92 ± 4.58	19.25 ± 5.69		**G × T: 3.594 (0.021)**	
	MSE(n = 13) ^d^	**19.92 ± 6.71**	24.77 ± 7.33*	**0.69**		
2 min step (rep)	NMSC(n = 11) ^a^	99.82 ± 15.94	103.45 ± 20.86		G: 1.907 (0.142)	
	NMSE(n = 12) ^b^	**102.08 ± 16.57**	120.33 ± 17.20**	**1.08**	**T: 20.088 (<0.001)**	
	MSC(n = 12) ^c^	94.67 ± 10.13	100.50 ± 20.10		G × T: 2.548 (0.068)	
	MSE(n = 13) ^d^	**95.92 ± 21.59**	116.62 ± 14.32**	**1.13**		
Sit & reach (cm)	NMSC(n = 11) ^a^	11.12 ± 8.53	11.25 ± 8.30		G: 0.484 (0.695)	
	NMSE(n = 12) ^b^	11.78 ± 5.07	13.94 ± 4.79		T: 3.514 (0.067)	
	MSC(n = 12) ^c^	12.21 ± 5.51	12.46 ± 5.10		G × T: 0.972 (0.415)	
	MSE(n = 13) ^d^	9.62 ± 7.04	10.60 ± 6.37			
TUG (sec)	NMSC(n = 11) ^a^	7.12 ± 1.27	6.50 ± 1.55		G: 2.232 (0.098)	
	NMSE(n = 12) ^b^	**6.35 ± 0.95**	5.34 ± 0.88***	**−1.1**	**T:28.220 (<0.001)**	
	MSC(n = 12) ^c^	6.86 ± 0.73	6.50 ± 0.91		G × T: 1.097 (0.361)	
	MSE(n = 13) ^d^	**6.52 ± 1.35**	5.75 ± 1.23**	**−0.6**		
F8WT (sec)	NMSC(n = 11) ^a^	26.69 ± 3.11	24.86 ± 3.81		G: 0.777 (0.513)	
	NMSE(n = 12) ^b^	**25.66 ± 5.88**	21.95 ± 3.86**	**−0.75**	**T: 19.723 (<0.001)**	
	MSC(n = 12) ^c^	27.81 ± 2.78	26.11 ± 5.26		G × T: 0.75 (0.528)	
	MSE(n = 13) ^d^	**27.46 ± 8.97**	24.09 ± 6.83**	**−0.42**		

Values are presented as Mean ± SD. a = NMSC, non-metabolic syndrome control group, b = NMSE, non-metabolic syndrome exercise group, c = MSC, metabolic syndrome control group, and d = MSE, metabolic syndrome exercise group. Statistical analysis was performed using the paired t-test: *p < .05; ***p < .001. Effect sizes cohen’d were interpreted small (d < 0.2), medium (d ≥ 0.5), and large (d ≥ 0.8).

Post hoc analyses with Cohen’s d effect sizes showed significant improvements in both exercise groups (NMSE and MSE). Grip strength (left and right) increased significantly (NMSE: p < 0.01 to 0.05, d = 0.46–0.72; MSE: p < 0.001 to 0.05, d = 0.28–0.38). Performance in the 30-second chair stand and 2-minute step improved significantly (all p < 0.05–0.01, d = 0.69–1.13). Functional performance measures, including TUG and F8WT, showed significant reductions in completion time (all p < 0.01 to 0.001, d = −1.10 to −0.42). No significant changes were observed for sit-and-reach (all p > 0.05).

## 4 Discussion

This study investigated the effects of a 10-week combined exercise intervention on circulating FGF21, adiponectin, and the FGF21/adiponectin ratio in older women with different metabolic syndrome statuses. The main findings of this study were as follows. First, key components of the FGF21–adiponectin axis were significantly modulated by the 10-week combined exercise intervention. Specifically, in the non-metabolic syndrome exercise group, both FGF21 and adiponectin significantly increased, whereas in the metabolic syndrome exercise group, FGF21 decreased while adiponectin increased. Consequently, the FGF21/adiponectin ratio significantly decreased in both exercise groups. Second, the exercise intervention improved body composition, physical fitness, blood lipid profiles, insulin resistance, and inflammatory markers in older women. These findings suggest that combined exercise can improve metabolic health and positively modulate the FGF21–adiponectin axis in older women, although the pattern of FGF21 responses may vary depending on baseline metabolic status.

From the perspective of the FGF21–adiponectin axis, FGF21 binds to its receptor complex and stimulates adiponectin secretion, while adiponectin mediates downstream metabolic effects such as improved insulin sensitivity, enhanced lipid metabolism, and anti-inflammatory actions [5,8]. Therefore, the changes in these two hormones should be interpreted as coordinated responses within a metabolic regulatory axis rather than as independent phenomena. In the present study, the non-metabolic syndrome exercise group showed significant increases in both FGF21 and adiponectin after exercise. This pattern suggests that in metabolically healthy older women, regular exercise may stimulate physiological FGF21 secretion while simultaneously promoting adiponectin release, thereby contributing to improved metabolic homeostasis. These findings are consistent with the results reported by Saeidi et al. (2019), who observed increases in both FGF21 and adiponectin following exercise in healthy postmenopausal women [17].

In contrast, the metabolic syndrome exercise group demonstrated increased adiponectin but decreased FGF21 levels following the intervention. This divergent response may be explained by the phenomenon of FGF21 resistance commonly observed in metabolic disorders. In metabolic syndrome, reduced expression of FGF21 receptors in adipose tissue can impair FGF21 signaling, leading to compensatory elevations in circulating FGF21 levels [[23], [24], [25]]. Therefore, the observed reduction in FGF21 after exercise in the metabolic syndrome group may reflect a normalization of previously elevated compensatory FGF21 levels and improved FGF21 responsiveness. In other words, while exercise may induce adaptive increases in FGF21 in metabolically healthy individuals, it may reduce abnormally elevated FGF21 levels in metabolically impaired individuals. The simultaneous improvements in insulin, HOMA-IR, TNF-α, IL-6, and adiponectin observed in this study further support this interpretation. Similar findings have been reported by Astinchap et al. (2021), who showed that exercise training reduced circulating FGF21 levels while improving metabolic markers in individuals with metabolic dysfunction [16].

Recent studies have suggested that the FGF21/adiponectin ratio may represent a sensitive indicator of metabolic health reflecting the interaction between these two hormones. This ratio has been reported to correlate positively with BMI, waist circumference, triglycerides, hsCRP, and HOMA-IR, and negatively with HDL-C [14]. In the present study, the FGF21/adiponectin ratio significantly decreased in both exercise groups. Notably, in the non-metabolic syndrome exercise group, the ratio decreased despite increases in both FGF21 and adiponectin, reflecting a relatively greater increase in adiponectin. In the metabolic syndrome exercise group, the ratio decreased more markedly due to the simultaneous decrease in FGF21 and increase in adiponectin. These findings suggest that the FGF21/adiponectin ratio may serve as a more integrated indicator of metabolic adaptation to exercise than FGF21 alone. Consistent with this interpretation, Stine et al. (2023) demonstrated that aerobic exercise significantly reduced the FGF21-to-adiponectin ratio and improved metabolic parameters in patients with non-alcoholic steatohepatitis [26].

Significant improvements were also observed in blood lipid profiles and insulin resistance markers. In particular, HDL-C significantly increased following the exercise intervention, while insulin and HOMA-IR significantly decreased. These findings suggest that regular exercise improves insulin sensitivity and lipid metabolism in older women. Previous studies have reported that exercise enhances glucose uptake in skeletal muscle, reduces adiposity, and attenuates inflammatory responses, thereby improving insulin signaling efficiency [27]. Moreover, the significant reductions in TNF-α and IL-6 observed in this study indicate that regular exercise can effectively attenuate chronic low-grade inflammation in older women. Such reductions in inflammatory cytokines may represent adaptive metabolic responses associated with decreased adiposity and improved adipokine regulation [28].

Body composition and physical fitness are key indicators of health status and functional independence in older adults. In the present study, both exercise groups showed significant reductions in body fat mass, percent body fat, and waist circumference, along with increases in muscle mass following the intervention. Improvements were also observed in several functional fitness measures, including grip strength, the 30-s chair stand, the 2-min step test, TUG, and F8WT. These findings are further supported by accelerometer-based evidence showing that higher physical activity levels are associated with lower odds of metabolic syndrome, particularly in older adults [29]. Together, these findings are consistent with previous studies demonstrating that regular exercise reduces body fat and improves muscle function in postmenopausal and older women [[30], [31], [32]]. In contrast, body weight and BMI did not significantly change, which may reflect the simultaneous reduction in fat mass and increase in muscle mass following exercise training [33]. In addition, sit-and-reach performance did not significantly change, possibly because the exercise program in this study primarily emphasized strength and functional capacity rather than flexibility training [34].

Despite the physiological and clinical significance, this study is subject to several limitations. First, the exercise intervention consisted of a single program, and therefore the effects of different exercise modalities such as aerobic training, resistance training, or high-intensity interval training were not compared. Second, metabolic syndrome classification was based on a single diagnostic criterion, and future studies should consider multiple diagnostic frameworks to enhance generalizability. Third, this study focused on circulating biomarkers and did not directly measure tissue-level FGF21 receptor expression or related signaling pathways. Future research integrating molecular analyses, such as receptor expression and downstream signaling pathways, would provide deeper insights into the mechanisms underlying exercise-induced regulation of the FGF21–adiponectin axis. Despite these limitations, the present study demonstrates that although the direction of FGF21 responses differed according to metabolic syndrome status, both exercise groups exhibited increased adiponectin and reduced FGF21/adiponectin ratios. These findings support the role of combined exercise as an effective non-pharmacological strategy for improving metabolic health in older women.

## 5 Conclusion

This study demonstrates that a 10-week supervised exercise intervention elicited favorable changes in body composition, physical fitness, insulin resistance, and low-grade systemic inflammation in older women. Exercise was also associated with modulation of the FGF21–adiponectin axis, as evidenced by increases in adiponectin across groups and divergent FGF21 responses according to baseline metabolic status. Specifically, FGF21 increased in women without metabolic syndrome but decreased in those with metabolic syndrome, resulting in a consistent reduction in the FGF21/adiponectin ratio following the intervention. These findings indicate that exercise-induced adaptations in the FGF21–adiponectin axis are influenced by metabolic phenotype, while the reduction in the FGF21/adiponectin ratio may reflect a common adaptive response to exercise. Collectively, these results support the role of regular exercise as a non-pharmacological strategy for improving metabolic health in older women. Further investigation is warranted to elucidate the mechanistic basis and clinical relevance of these axis-specific adaptations.

## CRediT authorship contribution statement

Liangliang Li and Deokhwa Jeong: Conceptualization, Methodology, Investigation, Data curation, Formal analysis, and Writing – original draft preparation; Hyeongmo Jeong and Jiye Choi: Visualization and Data curation; Sunghwun Kang: Project administration, Supervision, and Writing – review & editing.

## Funding sources

This research received no external funding.

## Ethics approval

All procedures conducted complied with relevant guidelines and regulations. The study was reviewed and approved by the Institutional Review Board (IRB) of Kangwon National University for human subject research (KWNUIRB-2022-08-003).

## Declaration of Generative AI and AI-assisted technologies in the writing process

Not applicable.

## Data availability

The data supporting the findings of this study are available from the corresponding author upon reasonable request.

## Declaration of competing interest

The authors declare that there are no competing interests related to this work.
