# Supplementary material for: Exercise-induced change in FGF21 and adiponectin and their association with metabolic syndrome in older women: a randomized controlled trial
Source: J Nutr Health Aging. 2026 Jul 4;30(9):100923. doi: 10.1016/j.jnha.2026.100923 (PMC13348046; doi:10.1016/j.jnha.2026.100923)
Supplement: Supplementary file 1 [file mmc1.docx]

**Supplementary Table 1.** Changes in metabolic syndrome components among final participants

| **Groups** | | **WC(cm)**  **≥ 85cm** | | **SBP(mmHg)**  **≥ 130mmHg** | | **DBP(mmHg)**  **≥ 85mmHg** | | **TG(mg/dL)**  **≥ 150 mg/dL** | | **HDL(mg/dL)**  **< 50 mg/dL** | | **Glucose(mg/dL)**  **≥ 100 mg/dL** | | |
| --- | --- | --- | --- | --- | --- | --- | --- | --- | --- | --- | --- | --- | --- | --- |
|  |  | **Pre** | **Post** | **Pre** | **Post** | **Pre** | **Post** | **Pre** | **Post** | **Pre** | **Post** | **Pre** | **Post** |  |
| **Non-**  **Metabolic**  **Syndrome**  **Control** | **1** | X: 70.70 | X: 70.90 | O: 137 | O: 153 | X: 52 | X: 66 | X: 98 | X: 100 | X: 90.60 | X: 91.20 | O: 110 | O: 106 |  |
|  | **2** | X: 84.10 | X: 83.00 | X: 105 | X: 100 | X: 72 | X: 60 | X: 93 | X: 138 | X: 56.40 | X: 50.90 | O: 114 | O: 118 |  |
|  | **3** | O: 90.30 | O: 91.50 | X: 120 | O: 130 | X: 51 | X: 76 | X: 90 | O: 169 | X: 69.00 | X: 68.50 | X: 95 | X: 96 |  |
|  | **4** | X: 78.20 | X: 77.70 | O: 134 | X: 115 | X: 75 | X: 82 | X: 122 | X: 117 | X: 75.00 | X: 78.50 | O: 133 | O: 117 |  |
|  | **5** | O: 85.80 | X: 83.60 | X: 129 | O: 153 | X: 64 | X: 57 | X: 63 | X: 69 | X: 53.80 | X: 55.40 | O: 127 | O: 149 |  |
|  | **6** | O: 91.20 | O: 91.80 | X: 123 | O: 138 | X: 59 | X: 60 | X: 93 | X: 91 | X: 54.10 | X: 60.40 | O: 108 | O: 111 |  |
|  | **7** | X: 70.70 | X: 71.40 | X: 127 | X: 114 | X: 72 | X: 81 | X: 97 | X: 54 | X: 50.00 | X: 52.30 | O: 139 | O: 131 |  |
|  | **8** | X: 75.00 | X: 76.30 | O: 132 | X: 121 | X: 61 | X: 67 | X: 146 | X: 101 | X: 51.00 | X: 56.80 | O: 125 | O: 127 |  |
|  | **9** | X: 84.00 | X: 84.90 | X: 128 | X: 129 | X: 70 | X: 73 | O: 220 | O: 174 | X: 50.50 | O: 49.50 | O: 116 | O: 113 |  |
|  | **10** | X: 70.50 | X: 70.70 | O: 159 | O: 152 | X: 72 | X: 73 | X: 85 | X: 67 | X: 50.20 | O: 49.20 | O: 133 | O: 140 |  |
|  | **11** | X: 84.00 | X: 84.80 | O: 153 | O: 156 | X: 74 | X: 76 | X: 146 | X: 144 | X: 50.80 | X: 50.00 | X: 96 | X: 97 |  |
| **Non-**  **Metabolic**  **Syndrome**  **Exercise** | **1** | X: 80.00 | X: 79.50 | X: 128 | X: 121 | X: 83 | X: 69 | X: 146.00 | X: 99 | X: 51.20 | X: 50.90 | O: 130 | O: 111 |  |
|  | **2** | X: 82.90 | X: 82.60 | X: 114 | O: 157 | X: 66 | X: 79 | X: 126.00 | X: 85 | X: 68.00 | X: 77.90 | O: 121 | O: 117 |  |
|  | **3** | O: 93.70 | O: 91.80 | X: 124 | O: 131 | X: 76 | O: 94 | X: 141 | X: 110 | X: 72.20 | X: 84.90 | O: 131 | O: 127 |  |
|  | **4** | X: 81.10 | X: 80.20 | X: 129 | O: 139 | X: 58 | X: 69 | X: 103 | X: 83 | X: 60.00 | X: 62.00 | O: 120 | O: 132 |  |
|  | **5** | X: 72.80 | X: 71.10 | O: 148 | O: 144 | X: 79 | X: 78 | X: 116 | O: 158 | X: 88.70 | X: 90.90 | O: 116 | O: 118 |  |
|  | **6** | X: 79.90 | X: 76.60 | O: 144 | O: 157 | X: 54 | X: 74 | X: 70 | X: 60 | X: 58.10 | X: 60.10 | O: 103 | O: 103 |  |
|  | **7** | X: 72.10 | X: 71.70 | O: 140 | O: 158 | X: 61 | X: 66 | X: 48 | X: 53 | X: 88.90 | X: 86.20 | O: 127 | O: 121 |  |
|  | **8** | X: 72.00 | X: 71.00 | X: 125 | X: 121 | X: 79 | X: 83 | X: 102 | X: 135 | X: 51.60 | X: 56.40 | O: 139 | O: 115 |  |
|  | **9** | X: 78.30 | X: 76.30 | O: 142 | X: 128 | X: 68 | X: 69 | X: 120 | O: 167 | X: 50.50 | O: 49.20 | O: 126 | O: 125 |  |
|  | **10** | X: 84.10 | O: 84.30 | O: 149 | X: 115 | X: 84 | O: 91 | X: 148 | X: 108 | X: 51.20 | X: 54.20 | O: 119 | O: 103 |  |
|  | **11** | X: 84.80 | O: 87.20 | O: 144 | O: 146 | X: 76 | X: 70 | X: 88 | X: 85 | X: 60.20 | X: 63.60 | O: 128 | O: 120 |  |
|  | **12** | X: 79.20 | X: 77.60 | O: 153 | O: 158 | X: 64 | X: 78 | X: 142 | X: 114 | X: 61.00 | X: 62.80 | O: 105 | O: 117 |  |
| **Metabolic**  **Syndrome**  **Control** | **1** | O: 85.80 | X: 81.10 | O: 143 | O: 151 | X: 64 | X: 80 | X: 138 | X: 80 | X: 56.60 | O: 49.70 | O: 105 | O: 117 |  |
|  | **2** | O: 87.10 | O: 88.20 | O: 154 | O: 158 | X: 82 | X: 71 | O: 196 | O: 196 | O: 45.30 | O: 45.30 | O: 121 | O: 127 |  |
|  | **3** | O: 100.70 | O: 102.50 | O: 140 | O: 154 | X: 71 | X: 74 | O: 162 | X: 123 | O: 41.70 | O: 41.20 | O: 149 | O: 128 |  |
|  | **4** | O: 93.00 | O: 90.30 | O: 147 | O: 136 | X: 60 | X: 70 | X: 138 | X: 122 | O: 46.10 | O: 46.20 | O: 115 | O: 105 |  |
|  | **5** | O: 85.10 | X: 83.80 | O: 131 | X: 100 | X: 77 | X: 70 | X: 78 | X: 59 | X: 72.00 | X: 67.80 | O: 127 | O: 126 |  |
|  | **6** | O: 91.40 | O: 93.10 | O: 156 | O: 158 | X: 68 | X: 72 | X: 148 | X: 89 | X: 55.40 | X: 63.00 | O: 127 | O: 103 |  |
|  | **7** | O: 92.00 | O: 91.10 | O: 145 | O: 154 | X: 67 | X: 75 | X: 145 | X: 105 | O: 49.50 | X: 50.70 | O: 134 | O: 114 |  |
|  | **8** | X: 74.00 | X: 70.50 | O: 157 | O: 152 | X: 51 | X: 57 | X: 96 | X: 147 | O: 38.10 | O: 36.80 | O: 146 | O: 130 |  |
|  | **9** | X: 81.30 | X: 80.70 | O: 159 | X: 128 | X: 72 | X: 74 | O: 158 | O: 265 | X: 54.20 | X: 50.60 | O: 134 | O: 135 |  |
|  | **10** | X: 83.00 | O: 87.20 | O: 135 | O: 140 | X: 75 | X: 75 | O: 188 | O: 397 | O: 42.40 | O: 34.50 | O: 130 | O: 139 |  |
|  | **11** | O: 91.70 | O: 91.00 | O: 146 | O: 139 | X: 66 | X: 72 | X: 119 | X: 121 | X: 53.50 | X: 55.50 | O: 132 | O: 116 |  |
|  | **12** | O: 92.20 | O: 93.80 | O: 133 | O: 133 | X: 76 | O: 87 | O: 152 | O: 213 | O: 35.30 | O: 35.60 | O: 127 | O: 136 |  |
| **Metabolic**  **Syndrome**  **Exercise** | **1** | O: 93.30 | O: 94.10 | O: 139 | O: 153 | X: 64 | X: 64 | X: 144 | X: 141 | O: 47.10 | O: 48.10 | O: 128 | O: 118 |  |
|  | **2** | X: 81.30 | X: 82.50 | O: 143 | O: 148 | X: 55 | X: 65 | O: 164 | X: 104 | O: 46.90 | X: 59.40 | O: 133 | O: 129 |  |
|  | **3** | O: 86.20 | O: 85.20 | O: 158 | O: 155 | X: 78 | X: 75 | O: 221 | O: 154 | X: 70.40 | X: 66.60 | O: 138 | O: 113 |  |
|  | **4** | O: 87.60 | O: 85.60 | O: 134 | O: 150 | O: 85 | X: 73 | X: 119 | X: 133 | X: 58.50 | X: 58.90 | O: 135 | O: 147 |  |
|  | **5** | X: 78.80 | X: 77.70 | O: 151 | X: 123 | X: 67 | X: 75 | O: 161 | O: 158 | X: 51.40 | X: 60.80 | O: 161 | O: 148 |  |
|  | **6** | O: 88.80 | O: 86.10 | O: 157 | O: 152 | X: 61 | X: 64 | X: 129 | O: 177 | O: 49.60 | O: 48.50 | O: 109 | O: 110 |  |
|  | **7** | O: 94.70 | O: 94.50 | X: 115 | O: 130 | X: 78 | X: 74 | O: 223 | O: 229 | X: 57.10 | X: 58.80 | O: 132 | O: 120 |  |
|  | **8** | X: 79.40 | X: 78.80 | O: 132 | O: 138 | X: 55 | X: 67 | X: 119 | O: 178 | O: 42.90 | O: 41.20 | O: 140 | O: 157 |  |
|  | **9** | O: 88.00 | O: 85.20 | O: 147 | O: 156 | X: 76 | X: 76 | X: 123 | O: 204 | X: 56.00 | X: 62.00 | O: 122 | O: 117 |  |
|  | **10** | X: 82.00 | X: 80.40 | O: 153 | O: 150 | X: 52 | X: 73 | O: 159 | O: 183 | O: 46.70 | X: 51.00 | O: 156 | O: 167 |  |
|  | **11** | O: 91.00 | O: 90.70 | O: 151 | O: 132 | O: 92 | X: 76 | O: 169 | O: 152 | O: 31.40 | O: 33.80 | O: 144 | O: 130 |  |
|  | **12** | O: 97.00 | O: 96.30 | X: 129 | O: 153 | O: 99 | O: 95 | X: 122 | X: 105 | X: 52.70 | X: 54.90 | O: 113 | O: 103 |  |
|  | **13** | O: 96.60 | O: 93.10 | O: 137 | O: 141 | X: 73 | O: 96 | X: 98 | X: 68 | X: 54.80 | X: 63.80 | O: 122 | O: 116 |  |

WC, waist circumference; BMI, body mass index; SBP, systolic blood pressure; DBP, diastolic blood pressure; TG, triglyceride; HDL-C, high density lipoprotein cholesterol; O, Criteria for metabolic syndrome; X, Not meeting the criteria for metabolic syndrome.


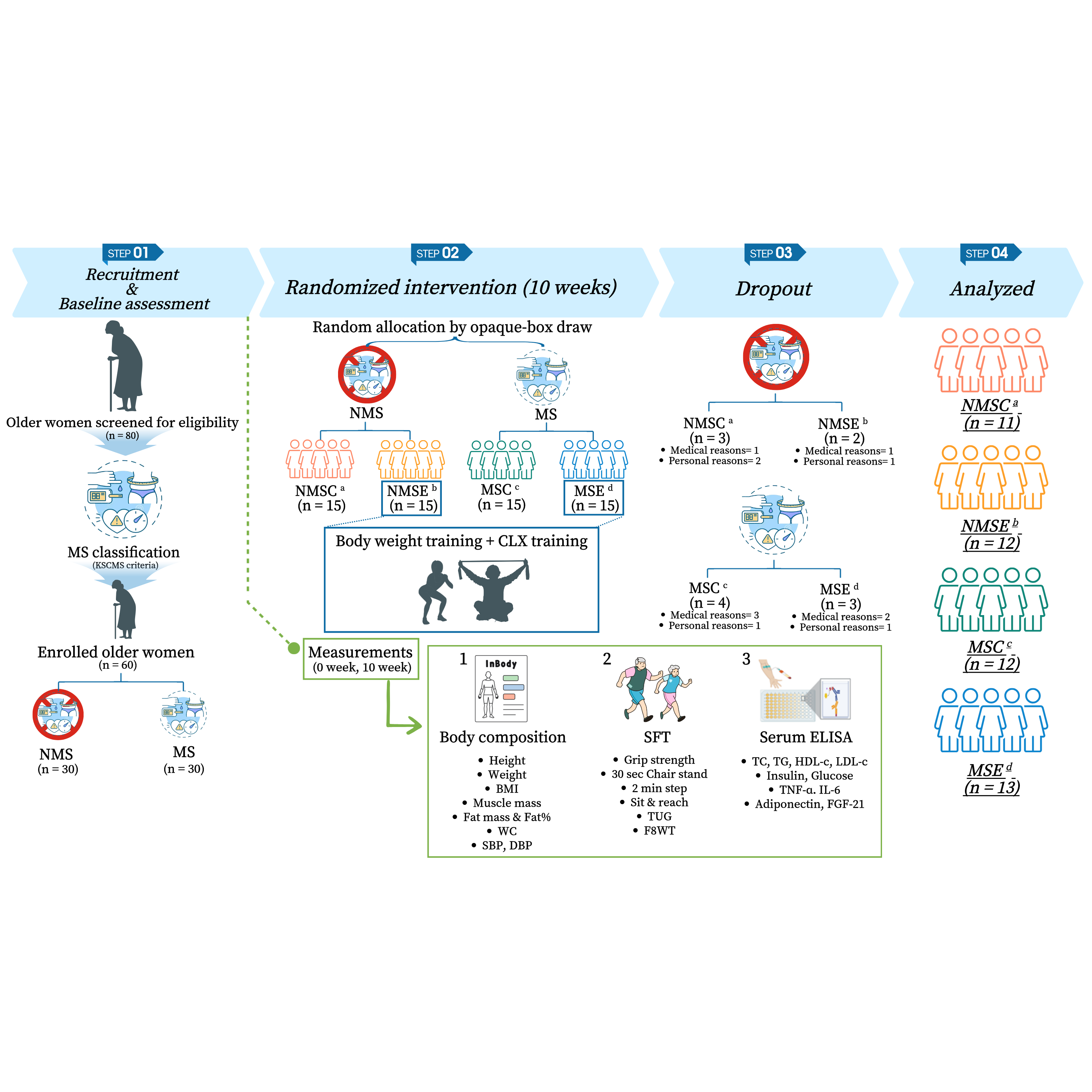


**Supplementary figure 1. Study design.** NMS, non-metabolic syndrome; MS, metabolic syndrome; NMSC, non-metabolic syndrome control; NMSE, non-metabolic syndrome exercise; MSC, metabolic syndrome control; MSE, metabolic syndrome exercise; KSCMS, korean society of cardiovascular and metabolic syndrome; BMI, body mass index; WC, waist circumference; SBP, systolic blood pressure; DBP, diastolic blood pressure; TUG, timed up and go; F8WT, figure of 8 walk test; ELISA, enzyme-linked immunosorbent assay; TC, total cholesterol; TG, triglyceride; HDL-C, high density lipoprotein cholesterol; LDL-C, low density lipoprotein cholesterol; TNF- ɑ, tumor necrosis factor-alpha; IL-6, interleukin-6; FGF21, fibroblast growth factor 21.
